# The Taxonomic Circumscription and Nomenclatural History of *Pilosella suecica* (Asteraceae): A Special Case of Grey Literature in Taxonomic Botany

**DOI:** 10.3390/plants13101301

**Published:** 2024-05-08

**Authors:** Alexander N. Sennikov

**Affiliations:** Botanical Museum, Finnish Museum of Natural History, University of Helsinki, 00014 Helsinki, Finland; alexander.sennikov@helsinki.fi

**Keywords:** exsiccata, hawkweeds, hybridisation, introgression, nomenclature, taxonomy, typification

## Abstract

The taxonomic history, nomenclature and application of the oldest species names available for the common hybrids between *Pilosella caespitosa* and *P. lactucella* are reviewed. Elias Fries created a nomenclatural and bibliographical collision when he replaced a printed label of his exsiccata *Herbarium normale* with its second version, distributed at a later date, in which the protologue of *Hieracium suecicum* had appeared. In this protologue, the new species name was validly published with a mere reference to the original description of *H. auricula* var. *majus*, thus being based on the type of the latter. In a later fascicle of the same exsiccata, Fries excluded this synonym and distributed a different morphotype of *H. suecicum*, which caused taxonomic confusion and re-description of the same taxon under the name *H. fennicum*. The surviving original material of *H. auricula* var. *majus* is rejected, and its neotype is designated, making *H. suecicum* the correct name for the hybrids strictly intermediate between *P. lactucella* and *P. caespitosa*. Such hybrids constitute the most common hybridogenous taxon of *Pilosella* in Scandinavia, Finland and neighbouring Russia, with many synonyms described from this area and partly typified here. Another hybridogenous taxon of the same origin, more similar to *P. lactucella* and previously known as *P. cochlearis*, is correctly named *P. stipitiflora* comb. nov. The nomenclatural value and bibliographic complexity of exsiccata, a commonly underestimated kind of grey literature in taxonomic botany, are further highlighted.

## 1. Introduction

Hybridisation is a common phenomenon in vascular plants. It has long been recognised as a fundamental evolutionary process [1,2], and many ancient taxonomic lineages may appear to have a hybrid background [3]. Hybridisation, as a process of reticulate evolution, commonly leads to alloploid speciation, which is often connected with polyploidy [4,5].

In contrast to hybridogenous speciation, which leads to the formation of novel evolutionary lineages, introgression is viewed as limited gene exchange between otherwise established species [6], which does not have an evolutionary effect other than changing fitness to the current and potential distribution environment, thus leading to local success and range expansions [7].

Interspecific hybridisation in *Pilosella*, disregarding ploidy levels, is extremely common, especially in some territories, and such hybrids are usually fertile [8,9]. The hybrids frequently appear, especially in secondary habitats, and may locally prevail over their parental taxa [9,10,11]. In *Pilosella*, the hybridisation includes both evolutionary scenarios, i.e., the formation of hybridogenous lineages [9] and introgression via repeated backcrossing [8]. Despite extensive experimental research on hybrid species complexes in *Pilosella* (e.g., [12,13,14,15]), morphological characters are considered more important than molecular markers in practical identification [16].

Type collections of the oldest species names in *Pilosella* have only recently been examined (e.g., [17]). In some cases, like *P. fallax* (Willd.) Arv.-Touv. [18] and *P. kalksburgensis* (Wiesb.) Soják [19], the earlier use (e.g., [20,21,22,23]) was abandoned because of misapplications. In other cases, like *P. acutifolia* (Vill.) Arv.-Touv. [24], the species name was transferred from one taxon (*P. glacialis* × *P. hoppeana*: [23]) to another (*P. brachiata* (DC.) F.W.Schultz and Sch.Bip.). Further nomenclatural perturbations of this kind can be expected in *Pilosella*, whose hybrid diversity in Europe counted the minimum of 122 hybridogenous “collective species”, i.e., hybrids between two or more (up to four) taxa that are considered non-hybridogenous [23]. So far, only a few of these taxa have been nomenclaturally evaluated.

Our study of the original collection of *P. floribunda* (Wimm. and Grab.) Fr. [25], which was recovered at the Komarov Botanical Institute, confirmed the historically predominant application of this species name to a triple hybrid between *P. caespitosa* (Dumort.) P.D.Sell and C.West s.l. (syn. *Hieracium pratense* Tausch), *P. lactucella* (Wallr.) P.D.Sell and C.West and *P. praealta* (Vill. ex Gochn.) F.Schultz and Sch.Bip. This lectotype agreed with the current use of the species name in Northern and Eastern Europe [22,26,27] and narrowed the broader taxonomic concept used in Central Europe [28].

The name *P. suecica* was incorrectly synonymised with the broadly defined *P. floribunda* on the belief that the latter is applicable to the binary hybrids between *P. caespitosa* and *P. lactucella* [28,29]. Another lumping concept, even more inclusive, subordinated *P. suecica* and *P. floribunda* to *P. caespitosa* on the assumption of their common hybrid origin [30]. With the lectotypification of *P. floribunda* [25], *P. suecica* became the correct name for the binary hybrid, and its lectotypification and synonymy are, therefore, of primary nomenclatural interest. This research is the main subject of the present contribution.

Similar taxa of the same hybrid origin, *P. fennica* and *P. cochlearis*, were recently accepted in some major taxonomic works and compilations [22,28]. Their identities and relationships with *P. suecica* are also clarified here.

Our previous work [31] has demonstrated that published exsiccata, a special kind of botanical publication and dissemination of information on plant diversity [32], have the utmost importance for the *Hieracium* nomenclature. The present contribution uncovers further aspects and complexities of this type of grey literature, which is still much under-explored and under-evaluated in plant taxonomy.

## 2. Results

### 2.1. Taxonomic Backbone

To characterise the non-hybridogenous species (*Pilosella caespitosa*, *P. lactucella*) and identify their hybrid parentage using morphology, the following diagnostic characters were used (Table 1), summarised from main published sources [22,29,33].

Diagnostic characters of the parental species were used for the inference of the hybrid origin already by Nägeli and Peter [34], and this approach is still considered useful in the diagnostic of *Pilosella* (cf. [25]). The most important diagnostic characters of *P. caespitosa* are a violet colouration of the stem base with abundant red-based hairs, dark green leaves with simple hairs on both surfaces and stellate pubescence on the lower side, and dark styles. The hybridisation with *P. lactucella* is recognisable through the yellowish-glaucous colour of leaves, which tend to be spathulate, undulate, glabrescent, with shorter and broader petioles, by low and slender stems, and by numerous slender glandular hairs in the synflorescence and along the stem; a prominent feature of this species is the sulphureous tint of its flowers, which is often recognisable in hybrids.

These characters are variously expressed in hybrids, with the dominance of diagnostic features of either of the parental species. Taking this dominance into account, three main variants can be distinguished among the hybrids between *P. caespitosa* and *P. lactucella*, which can be designated using hybrid formulas and binary names according to the standard adopted after Zahn’s monograph [20]: *P. caespitosa* < *P. lactucella*: *P. stipitiflora* (Nägeli and Peter) Sennikov; *P. caespitosa* × *P. lactucella*: *P. suecica*; *P. caespitosa* > *P. lactucella*: *P. colliniformis* (Nägeli and Peter) Dostál (Table 2).

In short, *P. colliniformis* is very similar to *P. caespitosa* but differs from the latter in the involucres with broad pale margins and in the leaves with a glaucous tint [33], which are partly subglabrous above. This morphotype is a hybrid with *P. lactucella*, which is most similar to *P. caespitosa* [34]. *Pilosella stipitiflora* closely resembles its other parent, *P. lactucella*, but differs in the greenish (vs. yellowish-glaucous) leaves and dark or black (vs. purely yellow) styles. It also differs from *P. suecica*, the intermediate variant, in its subglabrous (vs. ciliate along margins) leaves with the yellowish-glaucous (vs. greenish to green) colour, synflorescence branches with very few (if any) simple hairs and sulphureous (vs. yellow) flower colour.

The hybrids between *P. caespitosa* and *P. lactucella* are polymorphic, and there is no feasible limit between the members of this complex, neither in morphology nor in crossing barriers. However, their distinction stands for nearly 150 years, and its practical utility is therefore proven by time. The need for this taxonomic distinction is based not only on the contrasting morphology of the hybrids (some of them are hardly distinguishable from the parents by non-experts) but also on their different ecological preferences, usually coinciding with those of the parental species.

The correspondence between the historical applications of these hybrid names is given in Table 3. A broad variety of names used for these hybrids, also in various senses, indicate that the historical taxonomic circumscriptions in *Pilosella* were quite vague, and many (if not most) species names were variously misapplied and misinterpreted during their history. The nomenclature of *Pilosella* has been highly unstable, and no really established usage can be claimed in Europe.

### 2.2. Taxonomy and Nomenclature

#### 2.2.1. *Pilosella suecica* (Fr.) F.Schultz and Sch.Bip.

***Pilosella suecica*** (Fr.) F.Schultz and Sch.Bip. in Flora 45(27): 425 (1862). —*Hieracium auricula* var. *majus* Wahlenb., Fl. Upsal.: 261 (1820). —*Hieracium suecicum* Fr., Herb. Normale 9: No. 7, second version (1843–1845); Symb. Hist. Hierac.: 16 (1848). —*Hieracium auricula* var. *suecicum* (Fr.) Russow in Arch. Naturk. Liv-, Ehst- Kurlands, Ser. 2, Biol. Naturk. 3: 31 (1862). —*Hieracium floribundum* subsp. *suecicum* (Fr.) Nägeli and Peter, Hier. Mitt.-Eur. 1: 695 (1885). —*Pilosella floribunda* subsp. *suecica* (Fr.) Soják in Čas. Nár. Mus., Odd. Přír. 141(1–2): 45 (1972). —*Pilosella caespitosa* var. *suecica* (Fr.) T.Kukk in Kuusk et al., Fl. Baltic Countries 3: 97 (2003).

**Figure 1:**
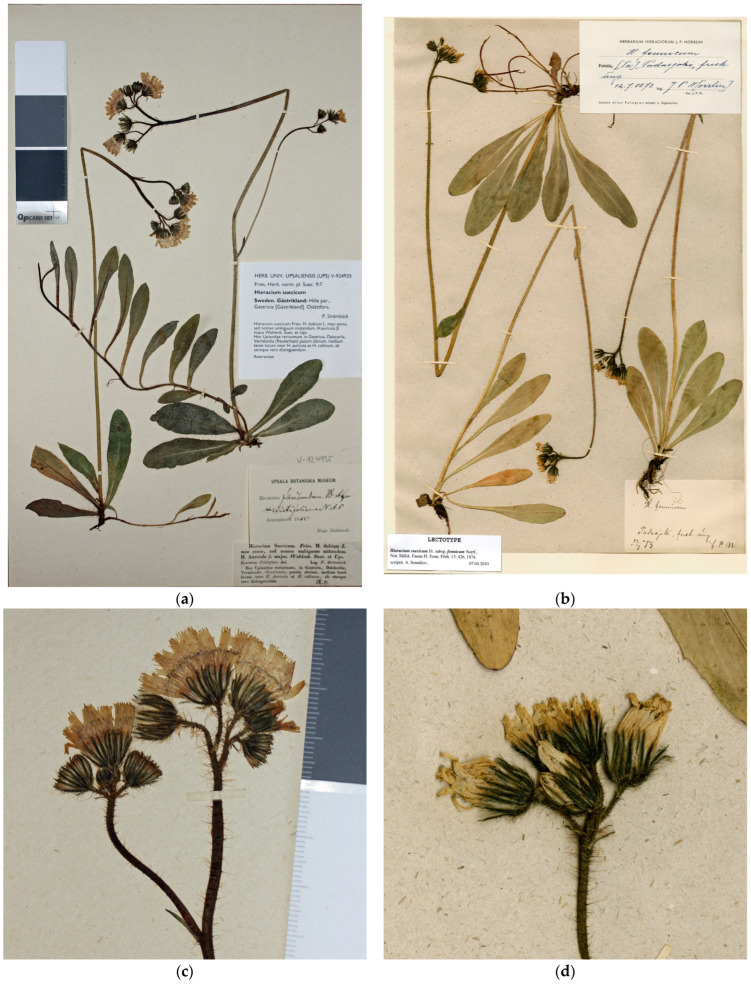
Type specimens of *Pilosella suecica* and its synonyms: (**a**) *Hieracium auricula* var. *majus* Wahlenb. =*H. suecicum* Fr., neotype sheet; (**b**) *H. suecicum* subsp. *fennicum* Norrl., lectotype sheet; (**c**) *H. auricula* var. *majus*, inflorescence; (**d**) *H. suecicum* subsp. *fennicum*, inflorescence.

Type (Figure 1a,c): Sweden. “Gestricia [Gästrikland], Oslättfors,” 07.1842, *P. Strömbäck* [E. Fries, Herbarium Normale 9: 7] (UPS V-924935, neotype designated here; isoneotypes H, LE, S, UPS V-104897 and V-107761 etc.).

=*Hieracium suecicum* subsp. *fennicum* Norrl. in Not. Sällsk. Fauna Fl. Fenn. Förh. 13: 426 (1874). —*Hieracium fennicum* (Norrl.) Mela, Lyhyk. Kasvioppi Kasvio: 74 (1877). —*Pilosella fennica* (Norrl.) Norrl. in Acta Soc. Fauna Fl. Fenn. 2(4): 107 (1884). —*Hieracium spathophyllum* subsp. *fennicum* (Norrl.) Nägeli and Peter, Hier. Mitt.-Eur. 1: 390 (1885). —*Hieracium longiscapum* subsp. *fennicum* (Norrl.) Zahn in Engler, Pflanzenr. 82: 1293 (1923).

Type (Figure 1b,d): Finland. Tavastia australis, “Padasjoki, frisk äng”, 12.07.1873, *J.P. Norrlin* (H, lectotype designated here).

=*Pilosella suecica* subsp. *cochlearis* Norrl. in Acta Soc. Fauna Fl. Fenn. 2(4): 173 (1884). —*Hieracium cochleare* (Norrl.) Norrl. in Mela, Lyhyk. Kasvioppi Kasvio, ed. 2, 2: 210 (1884), nom. illeg., non Huter (1874). —*Hieracium suecicum* subsp. *cochleare* (Norrl.) Hjelt, Fört. Finl. Fröv. Ormb.: 13 (1884). —*Pilosella cochlearis* (Norrl.) Soják in Preslia 43(2): 184 (1971).

**Figure 2:**
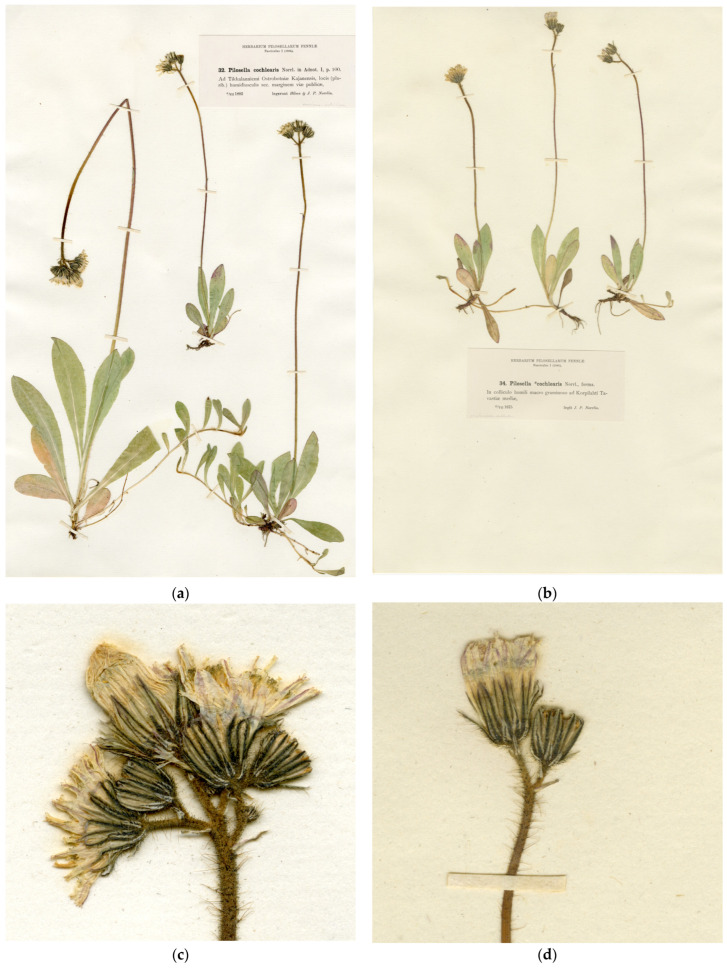
Type specimens of *Pilosella suecica* and its synonyms: (**a**) *Pilosella suecica* subsp. *cochlearis* Norrl., lectotype sheet; (**b**) *Hieracium floribundum* subsp. *cochleatum* Naeg. and Peter, lectotype sheet; (**c**) *P. suecica* subsp. *cochlearis*, inflorescence; (**d**) *H. floribundum* subsp. *cochleatum*, inflorescence.

Type (Figure 2a,c): Finland. “Ad Tikkalanniemi Ostrobotniae Kajanensis, locis (plurib.) humidiusculis sec. marginem viae publicae”, 04.07.1883, *H. Norrlin* and *J.P. Norrlin* [J.P. Norrlin, Herbarium Pilosellarum Fenniae 1: 32] (H, lectotype designated here; isolectotypes H, LE, O, S).

=*Hieracium floribundum* subsp. *cochleatum* Naeg. and Peter, Hier. Mitt.-Eur. 1: 700 (1885). —*Hieracium suecicum* subsp. *cochleatum* (Naeg. and Peter) Norrl. in Acta Soc. Fauna Fl. Fenn. 3(4): 34 (1888). —*Hieracium cochleatum* (Naeg. and Peter) Norrl., Hier. Exs. 1: ind. (1888).

Type (Figure 2b,d): Finland. “In colliculo humili graminoso ad Korpilahti Tavastiae mediae”, 13.07.1875, *J.P. Norrlin* [J.P. Norrlin, Herbarium Pilosellarum Fenniae 1: 34] (H, lectotype designated here; isolectotypes H, LE, O, S).

=*Hieracium floribundum* subsp. *ciliatifolium* Nägeli and Peter, Hier. Mitt.-Eur. 1: 699 (1885). —*Hieracium cochleatum* subsp. *ciliatifolium* (Nägeli and Peter) Zahn in Engler, Pflanzenr. 82: 1298 (1923).

**Figure 3:**
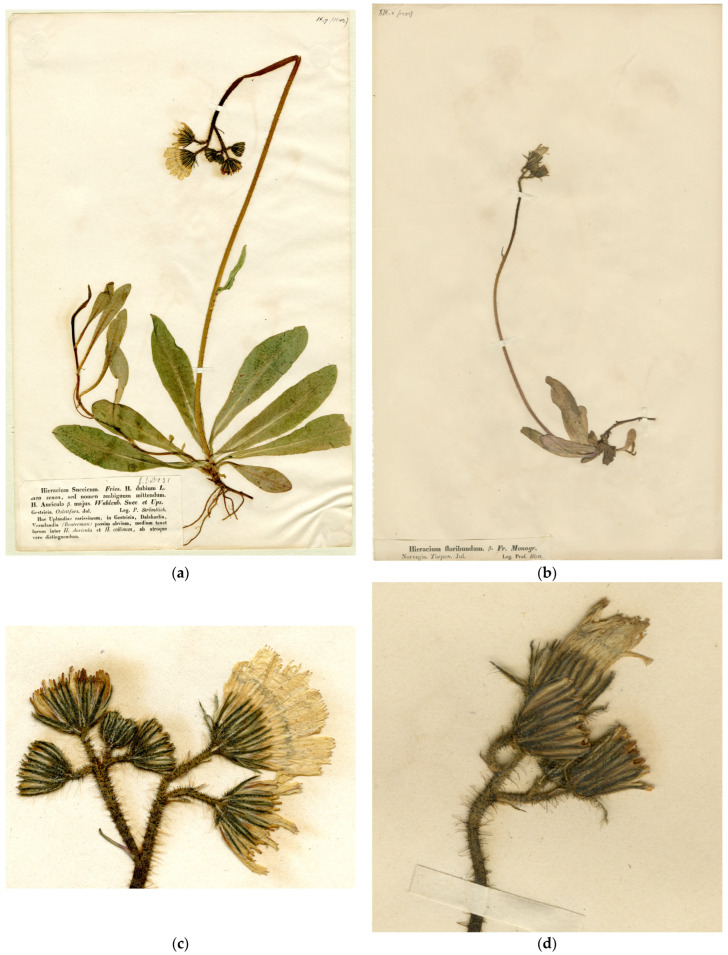
Type specimens of *Pilosella suecica* and its synonyms: (**a**) *Hieracium floribundum* subsp. *ciliatifolium* Nägeli and Peter, lectotype sheet; (**b**) *H. floribundum* subsp. *scissum* Naeg. and Peter, lectotype sheet; (**c**) *H. floribundum* subsp. *ciliatifolium*, inflorescence; (**d**) *H. floribundum* subsp. *scissum*, inflorescence.

Type (Figure 3a,c): Sweden. “Gestricia [Gästrikland], Oslättfors”, 07.1842, *P. Strömbäck* [E. Fries, Herbarium Normale 9: 7] (H, lectotype designated here; isolectotypes LE, S, UPS, etc.).

=*Hieracium floribundum* subsp. *scissum* Nägeli and Peter, Hier. Mitt.-Eur. 1: 698 (1885). —*Hieracium scissum* (Nägeli and Peter) Brenner in Acta Soc. Fauna Fl. Fenn. 12(1): 30 (1894). —*Hieracium cochleatum* subsp. *scissum* (Nägeli and Peter) Zahn in Engler, Pflanzenr. 82: 1296 (1923).

Type (Figure 3b,d): Norway. “Torpen”, 07.1847, *A. Blytt* [E. Fries, Herbarium Normale 13: 8] (H, lectotype designated here; isolectotypes LE, S, UPS, etc.).

*Stems*: 30–60 cm tall, erect, firm; lowermost internode short, pale to deep violet; with numerous simple hairs up to 3.5 mm long and with little or without stellate pubescence below, with scattered glandular hairs 0.2–0.4 mm, rare simple hairs and very lax stellate pubescence under the inflorescence. *Creeping stolons*: short or rather long, with spathulate leaves almost lacking stellate pubescence on the lower surface. *Leaves*: pale to intensely green, basal in a rosette; rosulate 5–13 × 0.9–1.2 cm, spathulate to oblong, shortly acute, shortly narrowed towards the base, with spiculiform teeth; cauline 1–2 (mostly in the basal part of the stem), like the basal but smaller and less narrowed to the base; all glabrous above, with some rigid, violet-based simple hairs along the middle nerve and the margin and solitary to rare stellate hairs along the middle nerve beneath, uppermost subglabrous. *Inflorescence*: corymbiform, usually compact, with 7–10 capitula; *branches*: rather firm, with abundant blackish glandular hairs 0.3–0.5 mm long, solitary simple hairs 2–2.5 mm long and dense stellate indumentum. *Involucral bracts:* inner 7–8.5 × 0.9–1.1 mm, olive or blackish-green with dark margins, broadly acute at the apex, with rare thin dark simple hairs ca. 2 mm long, rather dense thin black glandular hairs 0.5–0.6 mm long and scarce stellate *pubescence* throughout. *Flowers*: 12–13 mm long; ligules yellow. *Styles*: dark or pale dark. *Achenes*: ca. 2 mm long.

Ecology: Natural and disturbed meadows and grasslands in secondary habitats (roadsides, yards, and pastures).

Distribution: Native in Central and Northern Europe [28]; in Finland northwards up to *Ostrobottnia ultima* and *Regio kuusamoensis*, abundant [29]; in Eastern Europe northwards up to Russian Karelia [22,37]. Alien outside Europe: in Russian Siberia [38] and North America [39].

#### 2.2.2. *Pilosella stipitiflora* (Nägeli and Peter) Sennikov

***Pilosella stipitiflora*** (Nägeli and Peter) Sennikov, **comb. nov.** —*Hieracium floribundum* subsp. *stipitiflorum* Nägeli and Peter, Hier. Mitt.-Eur. 1: 699 (1885). —*Hieracium suecicum* var. *stipitiflorum* (Nägeli and Peter) Brenner in Acta Soc. Fauna Fl. Fenn. 9 (5): 39 (1893). —*Hieracium stipitiflorum* (Nägeli and Peter) Brenner in Acta Soc. Fauna Fl. Fenn. 12 (1): 30 (1894). —*Hieracium cochleatum* subsp. *stipitiflorum* (Nägeli and Peter) Zahn in Engler, Pflanzenr. 82: 1296 (1923).

**Figure 4:**
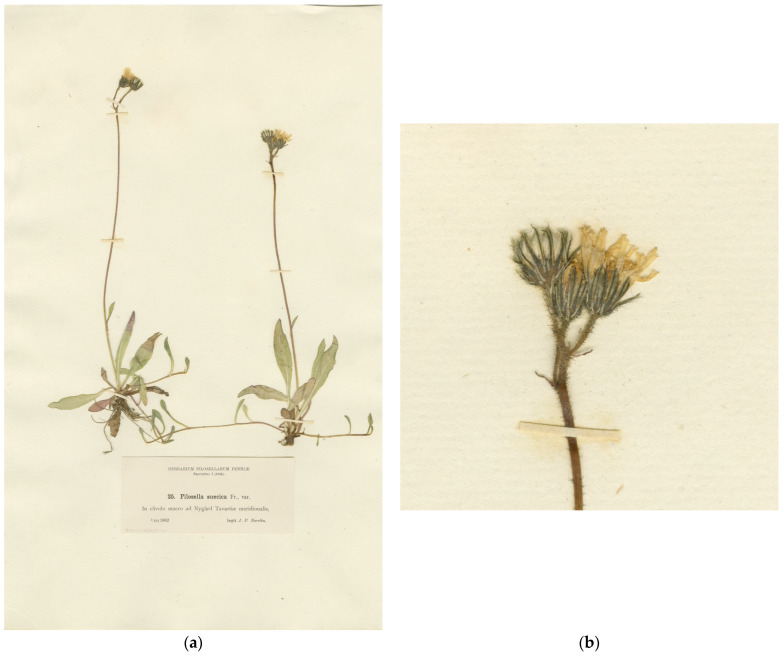
Type specimens of *Pilosella stipitiflora* (Nägeli and Peter) Sennikov (*Hieracium floribundum* subsp. *stipitiflorum* Nägeli and Peter): (**a**) lectotype sheet; (**b**) inflorescence.

Type (Figure 4): Finland. “In clivulo macro ad Nygård Tavastiae meridionalis”, 03.07.1882, *J.P. Norrlin* [J.P.Norrlin, Herbarium Pilosellarum Fenniae 1: 25] (H, lectotype designated here; isolectotypes H, LE, O, S).

*Stems*: 15–25 cm tall, erect, slender, pale green; with few to scattered simple hairs (dark-based in the lower part) and very sparse stellate pubescence, with numerous glandular hairs 0.2–0.4 mm. *Creeping stolons*: short or rather long, with narrowly spathulate leaves lacking stellate pubescence on the lower surface. *Leaves*: yellowish-green or pale green, basal in a rosette; rosulate 3–10 × 0.5–1 cm, narrowly spathulate or oblanceolate, obtuse, gradually narrowed to a broad or narrow petiole, with spiculiform teeth; cauline 1–2 (mostly in the basal part of the stem), like the basal but smaller and less narrowed to the base; all glabrous except for the basal part, with some rigid simple hairs along the middle nerve and the margin. *Inflorescence*: loosely corymbiform or irregularly branched, with 3–7 capitula; *branches*: slender, with abundant blackish glandular hairs 0.2–0.4 mm long and with or without solitary simple hairs and thin stellate indumentum. *Involucral bracts*: inner 6–7 × 0.8–1 mm, olive-green with pale margins, broadly acute at the apex, with few to sparse thin dark simple hairs 1–2 mm long, rather dense thin black glandular hairs 0.4–0.5 mm long and scarce stellate pubescence throughout. *Flowers* 10–11 mm long; ligules sulphureous. *Styles* pale dark or dark. *Achenes*: ca. 2 mm long.

Ecology: Natural and disturbed meadows and grasslands in secondary habitats (roadsides, yards and pastures).

Distribution: As for *Pilosella suecica*. This hybrid often occurs in the presence of *P. lactucella*.

## 3. Discussion

### 3.1. Confusing History of Hieracium suecicum

Fries [35] separated *Hieracium suecicum* Fr. and placed it between *Hieracium auricula* L. (now *Pilosella lactucella*) and *Hieracium floribundum* (*Pilosella floribunda*). According to our comparisons of his diagnostic characters (Table 4), Fries differentiated this new species from *P. lactucella* using flat, broader, obovate leaves (vs. undulate, narrower, ligulate), larger corymbose inflorescence and prominently dark styles. *Pilosella floribunda* was said to differ from the new species by narrower (lanceolate or spathulate) leaves and yellowish styles; this description was based on the plants belonging to *P. floribunda* in the sense of its lectotype, i.e., hybrids with *P. praealta* [25].

**Figure 5:**
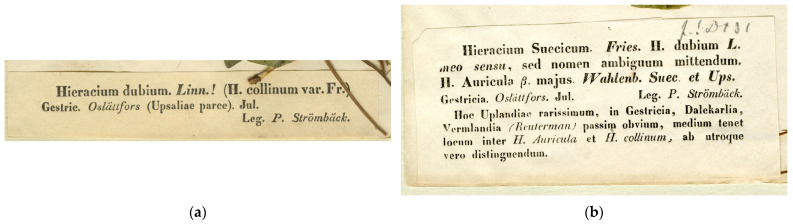
Elias Magnus Fries, Herbarium normale 9: 7, printed labels: (**a**) First version, distributed in 1843; (**b**) Second version, distributed in 1843–1845. Source: University of Helsinki.

In 1843, Fries [40] distributed a specimen from eastern Sweden under the name “*Hieracium dubium* L.”, which appeared on its printed label (Figure 5a) and in the index to the exsiccata set. This set was distributed to main herbarium institutions in the first turn, e.g., to Helsinki (pers. obs.) and Berlin [34]. Afterwards, Fries found this species name inappropriate because of its ambiguous application. He decided that this taxon should be recognised as a new species, which he named *H. suecicum*. To reflect this name change, between 1843 and 1845, Fries [41] printed and distributed a new version of the same label (Figure 5b), in which the new species name appeared and was accompanied by a synonym, “H. Auricula β. majus. Wahlenb. Suec. et Ups.” This reference to the previously published descriptions of *H. auricula* var. *majus* Wahlenb., including its protologue [42,43,44], makes the new species name validly published as a replacement name for this variety (Art. 6.12 [45]), thus based on the nomenclatural type of the latter (Art. 7.4 [45]). As the protologue of *H. suecicum* was published in the most unusual and obscure place (a replacement label for the exsiccata, which was present only in the sets distributed after the name change, in a limited number of copies available in herbarium collections rather than libraries), it attracted very scarce attention from taxonomic researchers (e.g., [46]), most of whom are still used to give credit for the valid publication of this species name to the later monograph [35]. The exact publication time of this label is uncertain: it postdates the original distribution of the exsiccata set [40], in which the old name appeared, but predates a checklist of Scandinavian plants by Fries [47], in which this label was cited under the changed name.

In the protologue of *H. suecicum*, Fries [41] also referred to “*H. dubium* L. meo sensu, sed nomen ambiguum mittendum”. This text is not a nomenclatural reference (because Fries abandoned *H. dubium* as an ambiguous and therefore unusable name and did not include it as a nomenclatural synonym) but a reference to his earlier [40] misapplication for the same plant. Similarly, this synonym was cited *pro parte* (regarding Swedish plants only, i.e., excluding foreign synonyms, one of which subsequently provided a lectotype of this species name [17]) in the first *Hieracium* monograph [35]. For this reason, *H. suecicum* is not a superfluous and illegitimate name as erroneously believed by Bräutigam and Greuter [28].

Fries incorporated the new taxonomy into his checklist of Scandinavian vascular plants and cryptogams [47] and his first monograph on the taxonomy of *Hieracium* worldwide [35]. Other researchers (e.g., [48]) adopted the change, using the specimen in the exsiccata as a taxonomic reference. But, after a few years, Fries changed his mind and noted that the plants of “*H. dubium*” in his earlier opinion [40] may be identical to *H. auricula* var. *majus*, which is not a synonym of *H. suecicum* that differs from the aforementioned variety in its yellow flowers with red stripes beneath (vs. sulphureous flowers in *H. auricula* = *P. lactucella*). Fries published the new circumscription of *H. suecicum* on herbarium labels in another issue of his exsiccata [49], adding a diagnosis of the flower colour. The plants that he distributed as the new “type” of *H. suecicum* were similar to the previous variant but demonstrated much greater morphological proximity to *P. lactucella* (stems slender, less hairy, leaves smaller, less greenish-coloured and much less hairy, flowering heads smaller).

Indeed, in his second monograph of *Hieracium*, Fries [36] confirmed the placement of the former *H. dubium* among the synonyms of *H. auricula* = *P. lactucella* and restored the variety *H. auricula* var. *majus*, but this variety was accepted in his own circumscription [50] for a vigorous variant of the species. The variety started its history from his earlier publication [51] rather than the actual protologue [42], but the varietal name was not validly published in that place because of the lack of a separate description.

In 1862, Fries [36] maintained *H. auricula* var. *majus* of Wahlenberg [42] as a partial synonym of *H. suecicum*, and interpreted it on the basis of its locality, Wahlenberg’s personal communication and herbarium collections. He did not refer to the suitability of the descriptions of the taxon in Wahlenberg’s works, probably because of their apparent mismatch: Wahlenberg [42,43,44] described plants with regularly hairy leaves, whereas Fries [35,36] stated that his *H. suecicum* has subglabrous leaves that are hairy only along the basal part of their margin. Fries’ description did not match the plants distributed in the first time [40], but perfectly agreed with the plants of the second distribution [49], which he considered typical of the species.

**Figure 6:**
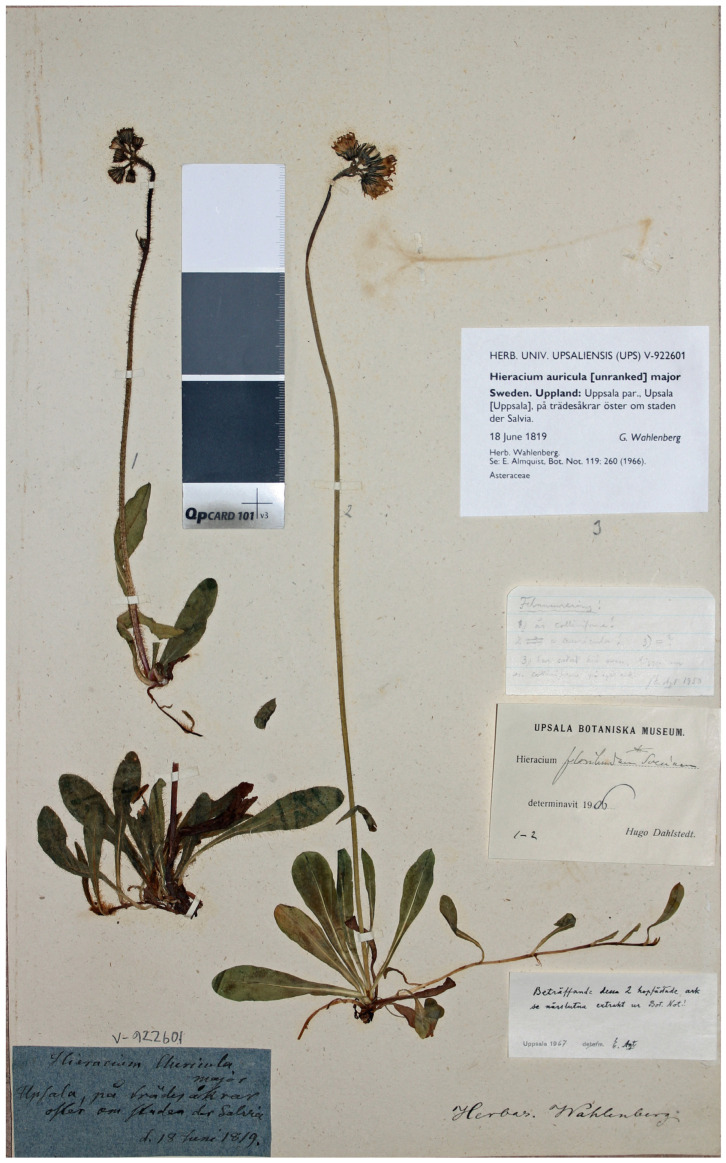
Surviving original material of *Hieracium auricula* var. *majus* Wahlenb., complete herbarium sheet (UPS V-922601).

As the varietal name *H. auricula* var. *majus* Wahlenb. provides the type of *H. suecicum* Fr., its typification is required to establish the nomenclatural application of the latter name. Almquist [52] examined the original material of Wahlenberg in the herbarium collections at Uppsala and reported that it contains three plants on a single herbarium sheet (Figure 6), two being complete and identifiable and the third one currently lacking flowers and therefore dismissed from consideration. The specimen (probably plant 2 in particular) was identified as *H. floribundum* subsp. *suecicum* (Fr.) Nägeli and Peter by H. Dahlstedt, the most renowned Swedish expert on *Hieracium* s.l. Almquist disagreed on this identity and assigned the first plant (with stems and leaves abundantly covered by long simple hairs) to *H. pratense* subsp. *colliniforme* (Peter) Zahn and the second plant (with stems and leaves subglabrous) to *H. auricula* = *P. lactucella* s.str. I partly agree with both interpretations in a way that the plants are referable to a hybrid complex between *P. lactucella* and *P. caespitosa* s.l., to which the name *P. suecica* belongs; the first plant represents a morphotype most resembling *P. caespitosa* (=*H. collinum* subsp. *colliniforme* Peter [34]), and the second plant is a variant more closely approaching *P. lactucella* (=*H. suecicum* or *H. floribundum* subsp. *suecicum* [34,36,49,53]).

A comparison of diagnostic characters stated in the protologue of *H. auricula* var. *majus* [42] with the same characters of preserved specimens in Herbarium Wahlenberg at UPS and the exsiccata distributed by Fries [40,49] (Table 5) unambiguously shows that Wahlenberg described the hybrids between *P. lactucella* and *P. caespitosa*, and the first interpretation by Fries [40] is matching the protologue. However, the second interpretation by Fries [49] is fully congruent with the second specimen in the original collection of Wahlenberg, which was indirectly referred to by Fries in 1862 [36]. Neither of the original specimens in Herbarium Wahlenberg agrees with the protologue, being in serious conflict with some major diagnostic characters used by Wahlenberg [42]: plant height (first specimen) or pubescence (second specimen). Specimens that are part of the original material but in conflict with the protologue cannot be used for lectotype designation because this type of choice can be superseded under Art. 9.19 [45]. Almquist [52] alluded that the first (hairy) specimen of the original material is an alien plant; it has, therefore, been collected by chance because of its unusual appearance. Similarly, the second (subglabrous) specimen may have been another chance collection that is linked with the protologue [45] but does not necessarily belong to the intended taxonomic circumscription. As no part of the available original material of *H. auricula* var. *majus* is suitable for lectotypification, a neotype may be designated under Art. 9.13 [45].

For the neotype designation, I propose a specimen distributed in the exsiccata [40] that was the first and most appropriate interpretation of the protologue. The neotype specimen (Figure 1a,c) has the most vigorous plants of the collection, with well-developed inflorescences and partly branched stems. Their leaves are ciliate along the margins and sparsely hairy on surfaces, in agreement with the original description of *H. auricula* var. *majus* in the protologue [42].

### 3.2. Early Synonyms of Hieracium suecicum

Norrlin [54] accepted the name *Hieracium suecicum* according to its reinterpretation [36,49], as a hybrid with the prominently dominating characters of *P. lactucella*. Ahead of the other Scandinavian authors, he held a narrow taxonomic concept and distinguished closely related and morphologically similar morphotypes of different hybrid origins. Since Fries shifted the circumscription of his *H. suecicum*, the hybrid between *P. caespitosa* and *P. lactucella* with intermediate morphology required a new name.

This hybrid was described as *H. suecicum* subsp. *fennicum* Norrl. [54] based on several specimens collected in three Finnish historical provinces: Tavastland (Häme), Savolaks (Savo) and Norra Karelen (Pohjois-Karjala), i.e., the central part of southern Finland. This name was quickly elevated to the species rank under *Hieracium* and *Pilosella* [53,55] and subsequently accepted in major taxonomic treatments [22,56]. A good specimen collected by Norrlin from Häme, his home province and main area of botanical research at that time, is designated here as lectotype (Figure 1b,d).

In his first detailed treatment of the Scandinavian *Pilosella*, Norrlin [53] provided a more detailed classification, in which he separated further subspecies and varieties from *P. suecica* and *P. fennica*. One of the new segregates, *P. suecica* subsp. *cochlearis* Norrl., was characterised by essentially the same characters as *P. fennica* in the previous works but deviated from the latter in smaller flowering heads. This segregate was accepted for the hybrid with the dominating characters of *P. lactucella* by some later authors (e.g., [23]), although its original specimens have the intermediate characters of their inflorescences and leaves. A specimen with larger plants distributed by Norrlin in his exsiccata [57] is designated here as lectotype (Figure 2a,c) to confirm this taxonomy. The specimens distributed by Norrlin in the later exsiccata [58] belong to the hybrid with the dominating characters of *P. lactucella* and may have had an influence on the subsequent acceptance of the species name in that sense.

Nägeli and Peter [34] published the most elaborated treatment of *Pilosella* in “Central Europe”, which, despite its restrictive title, included taxa from Scandinavia and Finland. They made extensive use of Norrlin’s exsiccata and accepted most of his taxa, albeit not necessarily in the same circumscription. They renamed Norrlin’s *P. suecica* subsp. *cochlearis* but distributed its syntypes between two subspecies, *H. floribundum* subsp. *suecicum* (Fr.) Nägeli and Peter and *H. floribundum* subsp. *cochleatum* Naeg. and Peter. The latter is not a nomenclatural replacement for *P. suecica* subsp. *cochlearis* because it included a reference to a single (and untypical) syntype of Norrlin’s subspecies name rather than to its protologue. For this reason, the subspecies name, corrected by Nägeli and Peter [34] to avoid later homonymy at the species rank, is the name of a new taxon with its own type material [31]; it is not a superfluous and, therefore, illegitimate replacement as incorrectly interpreted by Bräutigam & Greuter [28]. In agreement with this interpretation, its lectotype (Figure 2b,d) is designated from the exsiccata [57] cited in its protologue. This specimen also belongs to the hybrid with the intermediate morphology but is represented by small plants with depauperate inflorescences.

Contrary to the treatment of Bräutigam and Greuter [28], the combination *P. cochlearis* was not validly published by Norrlin, although it appeared in print in his taxonomic treatment [53] and on a single label in his exsiccata [57]. The definitive classification in these works was provided by Norrlin in the list of accepted taxa in the taxonomic treatment [53] (pp. 173–174) and in the index to the exsiccata [57], whereas the names appearing in the other parts of the text and on the label were provisional [31]. *Pilosella cochlearis* remained validly unpublished until its acceptance by Soják [59], who provided conditions for its valid publication and correctly noted that Norrlin did not accept the species.

Nägeli and Peter [34] included a set of Elias Fries’ *Herbarium normale* in their work. They used many specimens from these exsiccata to establish further new taxa. The specimens distributed as *H. dubium* [40] were named *H. floribundum* subsp. *ciliatifolium* Nägeli and Peter (Figure 3a,c), which is, therefore, a nomenclatural synonym of *H. suecicum* by our typification of the latter. The specimens distributed as *H. floribundum* [60] were described as *H. floribundum* subsp. *scissum* Nägeli and Peter (Figure 3b,d). These specimens do not belong to *H. floribundum* according to its common historical interpretation and our lectotype [25] but are referable to the hybrid with the intermediate morphology, which is named *H. suecicum* here, only slightly deviating towards *P. lactucella* by the shape of leaves. As the personal collection of Peter no longer exists [61], we designate lectotypes of both names from the exsiccata set at the University of Helsinki.

These lectotypifications provide the earliest synonyms of *P. suecica*. Further synonyms are expected from the Finnish *Hieracium* works [31] and elsewhere, but this synonymy requires a thorough inventory of the original material, which is still pending.

### 3.3. Pilosella stipitiflora Is the Correct Name for “P. cochlearis”

The hybrid between *P. caespitosa* and *P. lactucella*, which strongly resembles the latter parent, is morphologically distinct, and its recognition is, therefore, practical (cf. [22,23]. As the previously accepted names, i.e., *P. suecica* [22,34,53] or *P. cochlearis* [23], represent the morphologically intermediate hybrid, they are not suitable, and another name should be applied.

In the concept of Norrlin [53], *P. suecica* was accurately circumscribed to include plants resembling *P. lactucella*. Nägeli and Peter [34] separated a specimen distributed by Norrlin under the name *P. suecica* into a new subspecies, *H. floribundum* subsp. *stipitiflorum* Nägeli and Peter, which represented a minor deviation towards *P. caespitosa*. This taxon has been elevated to the species rank by Brenner [62] and currently provides the earliest legitimate name for this hybrid taxon. It is lectotypified here with a specimen from Norrlin’s exsiccata, and a new combination in *Pilosella* is effected to conform to the current taxonomy.

## 4. Materials and Methods

Historical herbarium collections are examined de visu at the University of Helsinki (H) and as digital images at the University of Uppsala (UPS). As the main *Hieracium* collections of Albert Peter have been destroyed [61], lectotypes of plant names established by this author are selected from the exsiccata cited in the protologues (sets kept at H). Lectotypes of plant names established by J.P. Norrlin are designated from his personal collection. Plant morphology, variability and distributions are evaluated on the basis of the herbarium collections at H and personal field observations.

The taxonomy of hybrid species of *Pilosella* follows the long-established tradition [19,20,22,23,34] in the acceptance of more than one species-level hybridogenous taxon between two parental species.

The nomenclature of the Finnish taxa is based on my previous inventory [31] with minor updates. Nomenclatural evaluations and decisions are based on the current International Code of Nomenclature for algae, fungi and plants [45]. Plant names are applied according to their nomenclatural types, except for *Pilosella caespitosa* (Dumort.) P.D.Sell and C.West, *P. colliniformis* (Nägeli and Peter) Dostál and *P. praealta* (Vill. ex Gochn.) F.Schultz and Sch.Bip., whose types are currently lacking, which are used in agreement with the main recent treatments [22,23,33].

## 5. Conclusions

The newly discovered protologue of *Hieracium suecicum* is based on *H. auricula* var. *majus*, whose original material is variable and not matching the protologue, but the designated neotype agrees with the original understanding and the current use of this species name in Northern and Eastern Europe. *Pilosella suecica* is the earliest correct name for all hybrids between *P. caespitosa* and *P. lactucella*, to which the name *P. floribunda* has been misapplied.

Numerous species names have been applied to various morphotypes resulting from the crosses between *P. lactucella* and *P. caespitosa* s.l., which demonstrate more or less intermediate morphology but may approach either of the parental species in their diagnostic characters. A few of such species names (*P. cochlearis*, *P. fennica*, *P. suecica*) have been recently in use by some authors [22,23]. I advise against the taxonomic recognition of such morphotypes because of their recurrent polytopic origin and the lack of morphological, ecological and biological delimitation.

A hybrid variant of *P. lactucella* with the introgression from *P. caespitosa* is morphologically close to *P. lactucella* but clearly differs from the latter in its spathulate and greenish-glaucous (vs. lingulate and yellowish-glaucous) leaves, more robust stems with scattered dark simple hairs (vs. very slender stems with few pale simple hairs), inflorescences corymbose (vs. loosely branched), involucres dark (vs. pale) and styles dark or black (vs. yellow). The taxonomic recognition of such plants is justified by their close morphological and ecological proximity to their parent (*P. lactucella*), which makes them dissimilar to the morphologically intermediate hybrids; yet they cannot be taxonomically treated as part of the species because of their interspecific hybrid origin. The correct name for these hybrids is *P. stipitiflora*. Such plants can be further placed in the *P. suecica* aggr. in the current classification of *Pilosella* [28].

The exsiccata published by Elias Fries, *Herbarium normale plantarum rariorum et criticarum Sueciae*, were standard reference for understanding taxonomic concepts in vascular plants of Scandinavia (with the neighbouring territories of Finland and Russia) in the 19th century. However, due to their old age and obscure bibliographic information, this exsiccata, as a nomenclatural reference, clearly belong to the corpus of grey literature, whose relevant inaccessibility and complexity may significantly hinder research in plant nomenclature [63]. Irregularities in their publication process have been already noted with the discovery of casual supplements [64]; the present contribution brings to light the very unusual practice of later re-issuing of the printed matter for individual numbers in this exsiccata, which may, as in the case of *Hieracium suecicum*, contain important nomenclatural novelties. Such a practice, if not properly explained and bibliographically deciphered, may mislead taxonomists and recorders to incorrect nomenclatural interpretations.

## Figures and Tables

**Table 1:** Diagnostic characters for *Pilosella caespitosa* s.l., *P. lactucella* and their hybrids with the intermediate morphology (*P. suecica*).

Taxon	*Pilosella lactucella*	*Pilosella suecica*	*Pilosella caespitosa*
stem	slender, 15–25 cm long, yellowish-green, with numerous short glandular hairs and scattered thin simple hairs	usually robust, 20–50 cm long, green or dark green, base red, with sparse to dense stiff simple hairs (red-based below)	robust, 25–40 cm long, dark green, base red, with dense stiff simple hairs (red-based below)
leaves	spathulate, gradually narrowed to a broad petiole, glaucous or yellowish-green, glabrous except for the basal part (margins and central nerve beneath long-ciliate)	obovate or oblanceolate, long-attenuate into a narrow petiole, pale or intensely green, margins ciliate, surfaces glabrous or sparsely hairy, some stellate pubescence below	oblanceolate, attenuate into a narrow petiole, dark green, margins ciliate, surfaces abundantly hairy, stellate pubescence below
inflorescence	branches with abundant short glandular hairs	branches with stiff glandular hairs and sparse to abundant long simple hairs	branches with stiff glandular hairs and abundant long simple hairs
phyllaries	6–7 mm long, with slender glandular hairs and single simple hairs	8–9 mm long, with stiff glandular hairs and rare simple hairs	8–9 mm long, with stiff glandular hairs and abundant simple hairs
flowers	sulphureous	yellow	yellow
styles	yellow	dark to black	black

**Table 2:** Diagnostic characters for *Pilosella stipitiflora*, *P. suecica* and *P. colliniformis*, the main variants of hybrids between *P. caespitosa* and *P. lactucella*.

Hybrid Taxon	*Pilosella stipitiflora* (*P. caespitosa* < *P. lactucella*)	*Pilosella suecica* (*P. caespitosa* × *P. lactucella*)	*Pilosella colliniformis* (*P. caespitosa* > *P. lactucella*)
stem	slender, 15–25 cm long, pale green, with numerous short glandular hairs and scattered simple hairs (dark-based below)	usually robust, 20–50 cm long, green or dark green, base red, with sparse to dense stiff simple hairs (red-based below)	robust, 25–45 cm long, dark green, base usually red, with abundant stiff simple hairs (red-based below)
leaves	spathulate or oblanceolate, gradually narrowed to a broad to narrow petiole, yellowish-green or pale green, glabrous except for the basal part (margins and central nerve beneath long-ciliate)	obovate or oblanceolate, long-attenuate into a narrow petiole, pale or intensely green, margins ciliate, surfaces glabrous or sparsely hairy, with some stellate pubescence below	oblanceolate, attenuate into a narrow petiole, dark green and slightly glaucous, margins ciliate, surfaces abundantly hairy (unevenly so above), with stellate pubescence below
inflorescence	branches with abundant short glandular hairs and possibly single simple hairs	branches with stiff glandular hairs and sparse to abundant long simple hairs	branches with stiff glandular hairs and abundant long simple hairs
phyllaries	6–7 mm long, with pale margins, with slender glandular hairs and sparse simple hairs	8–9 mm long, dark, with stiff glandular hairs and rare simple hairs	8–9 mm long, with broad pale margins, with stiff glandular hairs and abundant simple hairs
flowers	sulphureous	yellow	yellow
styles	(pale) dark	dark to black	black

**Table 3:** Nomenclature of main hybrids between *Pilosella caespitosa* and *P. lactucella* in major taxonomic works (as appeared in the cited works but with plant name authorship corrected).

Hybrid Taxon	*P. caespitosa* < *P. lactucella*	*P. caespitosa* × *P. lactucella*	*P. caespitosa* > *P. lactucella*
Fries, 1848 [35]	*H. suecicum* Fr., p.p.	*H. suecicum* Fr., p.p.; *H. floribundum* Wimm. and Grab. p.p.	–
Fries, 1862 [36]	*H. suecicum* Fr., sensu Fries (1862)	*H. floribundum* Wimm. and Grab. var.	–
Nägeli and Peter, 1885 [34]	*H. floribundum* subsp. *cochleatum* Nägeli and Peter; *H. auricula* subsp. *magnauricula* Nägeli and Peter	*H. spathophyllum* Peter	*H. collinum* subsp. *colliniforme* Peter
Zahn, 1923 [20]	*H. cochleatum* (Nägeli and Peter) Norrl.; *H. auricula* subsp. *magnauricula* Nägeli and Peter	*H. longiscapum* Boiss. and Kotschy ex Arv.-Touv.	*H. floribundum* Wimm. and Grab.; *H. pratense* subsp. *colliniforme* (Peter) Zahn
Sell and West, 1976 [33]	*H. lactucella* subsp. *magnauricula* (Nägeli and Peter) P.D.Sell	*H. floribundum* Wimm. and Grab.	*H. caespitosum* subsp. *colliniforme* (Peter) P.D.Sell
Schljakov, 1989 [22]	*P. suecica* (Fr.) F.Schultz and Sch.Bip., sensu Fries (1862)	*P. fennica* (Norrl.) Norrl.	*P. colliniformis* (Nägeli and Peter) Dostál
Bräutigam and Greuter, 2007 [23]	*P. cochlearis* (Norrl.) Soják	*P. floribunda* (Wimm. and Grab.) Fr.	*P. caespitosa* subsp. *colliniformis* (Peter) P.D.Sell and C.West
this work	*P. stipitiflora* (Nägeli and Peter) Sennikov	*P. suecica* (Fr.) F.Schultz and Sch.Bip.	*P. colliniformis* (Nägeli and Peter) Dostál

**Table 4:** Diagnostic characters of *Hieracium suecicum* and related species as used by Fries [35], with the author’s emphasis in Italics.

Characters/Species	*Hieracium auricula*	*Hieracium suecicum*	*Hieracium floribundum*
stolons	rhizomate repente stolonifero	rhizomate repente stolonifero	rhizomate repente substolonifero
basal leaves	*foliis lingulatis obtusis glaucis utrinque nudis* versus basin ciliatis	foliis obovatis planis obtusis glaucis utrinque nudis et glabris… folia laete glauca, versus basin tantum ciliata	*foliis* lanceolatis spathulatisque viridi-glaucescentibus margine carinaque l. utrinque setosis, *subtus rare flocculoso-stellulatis*, primariis obtusis
cauline leaves	scapo subunifolio	scapo submonophyllo	scapo monophyllo
inflorescences	scapo… apice cymoso	*corymbo composito* fastigiato	scapo… oligocephalo corymboso furcatove
inflorescence branches	*pedunculis simplicibus arcuato-adscendentibus*, furcatove	*ramis patentibus polycephalis*	sursum pedunculisque glanduliferis… *nigricante nigrohispidis*
phyllaries	squamis obtusis pallidis	involucris subnudis ovatis	cum *involucro ovato-globoso nigricante nigrohispidis*, squamis obtusis concoloribus
styles	stylo luteo glabro	*stylo fusco-hispidulo*	stylo luteo

**Table 5:** Diagnostic characters of *Hieracium auricula* and *H. auricula* var. *majus* [42], compared with those of herbarium specimens in the original collection of *H. auricula* var. *majus* (UPS) and the exsiccata of *H. suecicum* [40,49]. Matching characters of herbarium specimens in bold, mismatching ones in Italics.

Characters/Taxon or Specimen	*Hieracium auricula* s.str.	*Hieracium auricula* var. *majus*	Herb. Wahlenberg, Plant 1	Herb. Wahlenberg, Plant 2	Herbarium Normale IX: 7
stems	scapus plerumque spithamalis	scapis plusquam pedalibus supra medium bi-trifidis	*ca. 20 cm*, *unbranched*	**ca. 30 cm**, *unbranched*	**30–40 cm**, *unbranched* or **branched**
inflorescence	scapus bi-triflorus	ramis subumbellatis	**umbelliform, 6 flowering heads**	**umbelliform, 4 flowering heads**	**umbelliform, 5–7 flowering heads**
leaves	folia glabra et ad basin tantum ciliata	foliis ubique ciliatis	**hairy along margins and on both sides**	*hairy along margins at the base only*	**hairy along margins and sparsely so on both sides**
stolons	stolonibus repentibus	stolonibus plerisque scapiferis	*creeping*, *rudimentary*	*creeping*, *sterile*	*creeping*, *sterile*, sometimes **flowering**

## Data Availability

No new datasets were generated during this study. The herbarium specimens used in this work are publicly available at the Universities of Helsinki and Uppsala.
